# Prostate Cancer Presenting as Huge Mediastinal and Retroperitoneal Masses: Case Report and Review of the Literature

**DOI:** 10.1155/2017/7312740

**Published:** 2017-03-30

**Authors:** Safa Alshaikh, Zainab Harb

**Affiliations:** Department of Pathology, Salmaniya Medical Complex, Manama, Bahrain

## Abstract

Mediastinum and retroperitoneum are exceedingly rare sites for metastatic prostate cancer to occur. Here, we present the case of a 67-year-old male patient with incidental findings of mediastinal and retroperitoneal masses which were found to be due to metastatic prostate adenocarcinoma based on histopathology and immunohistochemical studies and later on supported by the significantly elevated Prostate Specific Antigen (PSA) levels. Prostate cancer should always be considered in the differential diagnosis of elderly men presenting with metastatic epithelial tumors even in unusual sites.

## 1. Introduction

Prostate carcinoma is the second most common malignant disease in men. It usually spreads by direct extension and also metastasizes to bone and lymph nodes of pelvis [1]. Our patient presented with mediastinal mass as the first manifestation of prostate cancer. Unusual prostate carcinoma metastases are usually encountered in the presence of known advanced disease but rarely as the first presentation. In one series, mediastinal adenopathy was reported as the initial presentation in only 0.8% of patients with prostate cancer [2]. Being aware of this unusual and rare presentation of metastatic prostate carcinoma might decrease diagnostic delay and allow earlier treatment.

## 2. Case Report

**Figure 1:**
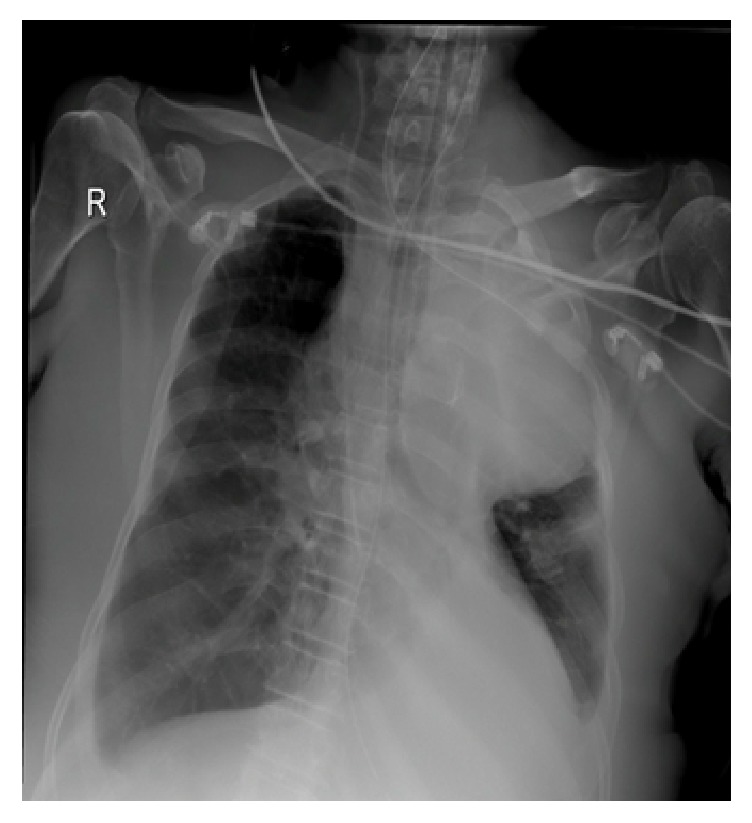
Chest X-ray shows left mediastinal mass.

**Figure 2:**
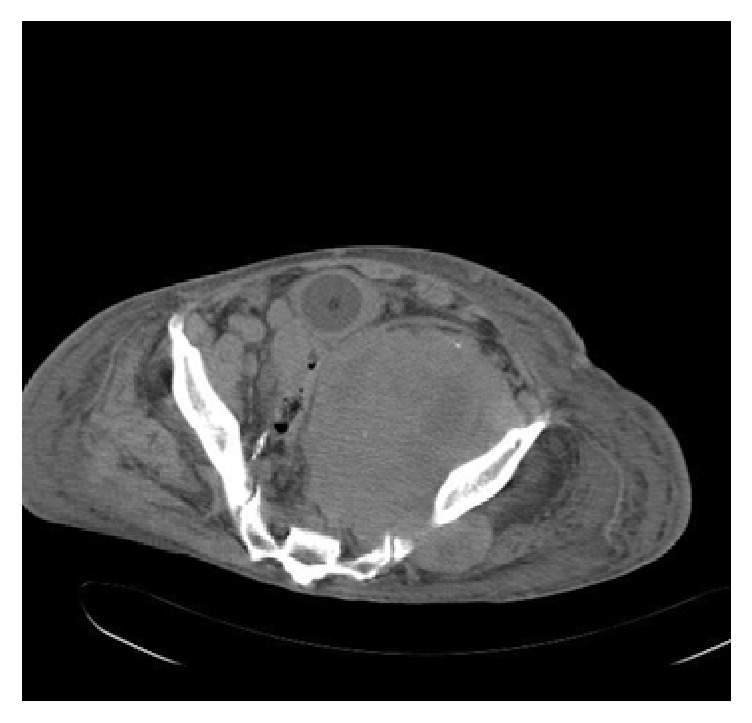
CT of abdomen and pelvis shows the retroperitoneal masses.

**Figure 3:**
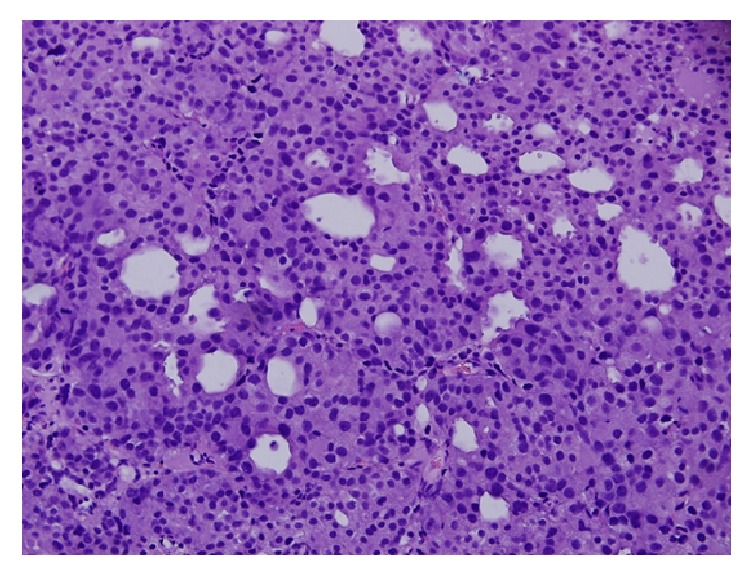
Hematoxylin and eosin (H&E) examination of the core needle biopsy of the mediastinal mass (×20).

**Figure 4:**
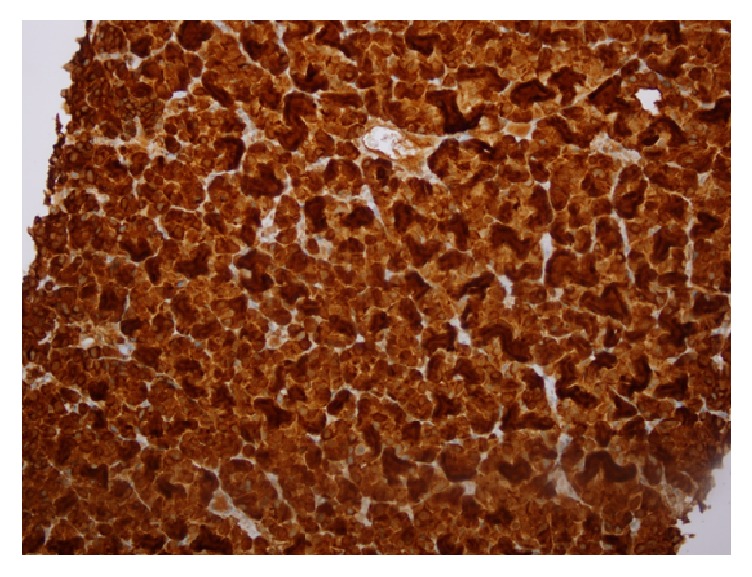
Immunohistochemical examination of the mediastinal mass core needle biopsy using the Prostate Specific Antigen (PSA) stain (×20).

A 67-year-old gentleman presented for the first time with acute urinary retention and left leg swelling. The urinary retention was managed by a urinary catheter. Ultrasound of the lower limbs revealed an extensive left lower limb deep venous thrombosis involving the left femoral vein. The patient was treated with anticoagulants and IVC filter. At that time, his Prostate Specific Antigen (PSA) level was around 77.2 ug/L, but no prostate biopsy was done. Six months later, he presented with shortness of breath and productive cough with production of whitish sputum for two days. Pulmonary embolism was ruled out after blood workup and imaging investigations. Nevertheless, his chest X-ray showed left sided chest mass (Figure 1). CT scan with oral contrast medium (intravenous contrast medium was not administrated due to impaired renal function) was also performed and showed lobulated mediastinal masses extending to the supraclavicular regions bilaterally, the largest measuring 9.8 cm in maximum dimension. There were also retroperitoneal masses measuring in combination 15 × 15 cm extending from upper abdomen down to the level of aortic bifurcation and associated with significant bilateral hydronephrosis and hydroureters (Figure 2). The radiological impression was of lymphoma. A core needle biopsy was taken from the mediastinal mass and showed epithelial tumor cells arranged in nesting pattern with focal acinar formation. The cells show enlarged nuclei and abundant eosinophilic cytoplasm with focal necrosis and scattered mitoses (Figure 3). Immunohistochemical stains were done and surprisingly showed diffuse and strong positivity for PSA (Figure 4) and CK8/18. The tumor was negative for CKAE1/AE3, NSE, CD45, PLAP, CD117, S-100, Chromogranin, Calretinin, TTF-1, CD34, and EMA.

The diagnosis was stage IV metastatic prostate carcinoma (pM1a). After the histopathology diagnosis was given, the clinician was advised to do PSA level. Serum PSA level was done and it was >1000 ug/L. No further investigations or followups were done because the patient travelled for treatment to his original country.

## 3. Discussion

Prostate cancer is the second most common noncutaneous malignant disease in men worldwide. Older age is the main risk factor, but, since the introduction of the serum Prostate Specific Antigen (PSA) as a screening test in the late 1980s, prostate cancer has been diagnosed at younger ages and earlier stages. At present, most cases of prostate cancer are diagnosed on prostate needle biopsies performed either for elevated serum PSA levels or for abnormal prostate on digital rectal exam [1, 3].

Less frequently, patients present initially with metastatic prostate cancer. Prostate carcinoma is known to metastasize by direct extension and lymphatic spread usually to regional pelvic lymph nodes [1–5]. It also disseminates hematogenously to lymph nodes outside the true pelvis (Mla disease as in our case), to the bone (M1b disease), and rarely to the liver and lung (M1c disease) [5–8].

Metastases in unusual sites are exceedingly rare. These include supraclavicular, cervical, mediastinal, pulmonary, and retroperitoneal metastases, and they are rarely the first clinical presentation of prostate cancer [4–9]. In a large series of mediastinal metastases, Cetin et al. reported that only 1% are of prostatic origin [8]. In addition, abdominal metastases have been reported only in isolated cases [4, 10].

Mediastinal masses can be challenging both clinically and pathologically. Various procedures can be used to reach a pathologic diagnosis. These include minimally invasive transthoracic or transbronchial fine needle aspiration cytology (FNAC) or core needle biopsy (CNB), mediastinoscopy, video-assisted thoracoscopy, and open surgical biopsy. Open surgical biopsy is often associated with significant morbidity. FNAC and CNB can be performed safely under imaging guidance without any discomfort to the patient and both have high diagnostic yields. Inadequate cytological material obtained by FNAC procedure sometimes delays the diagnosis and it prevents using it as a first-line diagnostic procedure. A larger tissue obtained by core needle biopsy allows more architectural, cytological, and immunohistochemical studies and increases the diagnostic accuracy. For our patient the clinician preferred to start with core needle biopsy rather than FNAC [11].

The main differential diagnoses of a malignant epithelial tumor in the mediastinum include primary thymic carcinoma (AE1/AE3 and CD117 positive), thyroid carcinoma (TTF1 positive), germ cell tumors (PLAP and CD117 positive), neuroendocrine tumors (NSE and Chromogranin A positive), mesothelioma (Calretinin and WT1 positive), and lymphoma (CD45 positive, although unlikely with this morphology). All these have been excluded in our case. Metastatic epithelial tumors from other sites should always be kept in the differential diagnoses after ruling out the primary tumors. These can be either of lung origin (TTF1 positive), gastrointestinal tract (CDX2 positive), prostate in males (PSA positive), breast in females (ER positive), and melanoma (S100 and HMB45 positive). Poorly differentiated carcinomas are known to be heterogeneous in their expression of antigens recognized by epithelial markers, so it is always wise to use multiple markers in the screening panel. In a study of 98 poorly differentiated carcinomas, cytokeratin and epithelial membrane antigen (EMA) combined detected epithelial differentiation in 99% of the cases, compared to 71% when each was used alone [12].

In conclusion, the main diagnostic challenge in this case was the initial presentation of prostate cancer as mediastinal and retroperitoneal masses. Although prostate cancer has been found to be responsible for only 2% of all metastatic carcinomas of unknown origin, it should always be suspected in any male patient older than 50 years especially if urologic symptoms and elevated PSA levels are also present [8].
